# The role of *Tannerella forsythia* and *Porphyromonas gingivalis* in pathogenesis of esophageal cancer

**DOI:** 10.1186/s13027-019-0220-2

**Published:** 2019-01-30

**Authors:** Bartosz Malinowski, Anna Węsierska, Klaudia Zalewska, Maya M. Sokołowska, Wiktor Bursiewicz, Maciej Socha, Mateusz Ozorowski, Katarzyna Pawlak-Osińska, Michał Wiciński

**Affiliations:** 1 Department of Pharmacology and Therapeutics, Faculty of Medicine, Collegium Medicum in Bydgoszcz, Nicolaus Copernicus University, M. Curie 9, 85-090 Bydgoszcz, Poland; 2 Department of Pathophysiology of Hearing and Balance System, Faculty of Medicine, Collegium Medicum in Bydgoszcz, Nicolaus Copernicus University, M. Curie 9, 85-090 Bydgoszcz, Poland; 3 Department of Obstetrics, Gynecology and Gynecological Oncology, Faculty of Medicine, Collegium Medicum in Bydgoszcz, Nicolaus Copernicus University, Ujejskiego 75, 85-168 Bydgoszcz, Poland

**Keywords:** Esophageal cancer, Tannerella forsythia, Porphyromonas gingivalis

## Abstract

*Tannerella forsythia* and *Porphyromonas gingivalis* are anaerobic*,* Gram-negative bacterial species which have been implicated in periodontal diseases as a part of red complex of periodontal pathogens. Esophageal cancer is the eight most common cause of cancer deaths worldwide. Higher rates of esophageal cancer cases may be attributed to lifestyle factors such as: diet, obesity, alcohol and tobacco use. Moreover, the presence of oral *P. gingivalis* and *T. forsythia* has been found to be associated with an increased risk of esophageal cancer. Our review describes the role of *P. gingivalis* and *T. forsythia* in signaling pathways responsible for cancer development. It has been shown that *T. forsythia* may induce pro-inflammatory cytokines such as IL-1β and IL-6 by CD4 + T helper cells and TNF-α. Moreover, gingipain K produced by *P. gingivalis,* affects hosts immune system by degradation of immunoglobulins and complement system (C3 and C5 components). Discussed bacteria are responsible for overexpression of MMP-2, MMP-2 and GLUT transporters.

## Background

Cancer is a significant problem in the modern world. It concerns the entire population. In developed countries, this is the second cause of death, after cardiovascular diseases, with a relatively high mortality rate. Over the past three decades, there has been a two-fold increase in the incidence of malignancies in Poland [1]. In 2018, according to the Global Cancer Observatory (GCO), 572,034 new cases of esophageal cancer have been reported for both sexes, while the number of deaths from esophageal cancer was 508,585. Currently, there are 185,630 new cases of illness and 113,388 deaths [2] in Poland. Among all cancers, esophageal tumors occupy the eighth position as to the incidence of the disease. Mild esophageal tumors are rare. More often, there are esophageal malignancies, which constitute 2% of all malignant tumors. There are two types of malignancies: squamous cell carcinoma (90% of cases) and adenocarcinoma (10%). Currently, there is a downward trend in the incidence of squamous cell carcinoma and an increase in adenocarcinoma [1]. Esophageal cancer is more common in men than in women. According to statistics, the incidence rate among men is 1%, and among women 0.3%. Esophageal cancer is more common in older people, usually in the 55–64 age group. The most common cause of esophageal cancer is smoking tobacco [1]. Other recognized factors predisposing to cancer development are: dietary factors, i.e. eating spicy foods, scalding hot foods or deficiencies of vitamins, i.e. riboflavin and nicotinic acid [3]. The most important prognostic factor of esophageal squamous cell carcinoma is the consumption of high-percentage alcohols. The factor contributing to the risk of adenocarcinoma development is gastroesophageal reflux disease (GERD), followed by Barett’s esophagus. It has been shown that in people with longer duration of gastroesophageal reflux disease, the risk of adenocarcinoma is about 5 times higher than in those without GERD [4].

Another important cause of adenocarcinoma is obesity. According to the study, patients with higher BMI are 3–7 times more exposed than people with normal body weight [5]. Esophageal carcinoma processes can be induced by some bacteria, such as *Porphyromonas gingivalis* and *Tanerella forsythia.*

## Characteristics of *Porphyromonas gingivalis*

*Porphyromonas gingivalis* was discovered in the 1980’s, but its participation in pathogenicity was not yet fully understood. Today, it is a well-known fact that it is one of the main etiological factors in the pathogenesis of periodontal disease [6]. The main environment for *P. gingivalis* development is the subgingival groove of the human oral cavity [7].

Bacteria of the *P. gingivalis* species stain red in the Gram method, which classifies them as Gram-negative. They are included in the Bacteroidetes cluster [7]. They are immobile, anaerobic bacteria in the shape of short sticks. In terms of biochemical features, they are indole-positive and do not have the ability to ferment sugars [8]. They require iron for their development [7]. They grow in the form of gray, small colonies with a diameter of approx. 1 mm. On the culture medium, they can be observed after 48 h of incubation [9]. On blood agar, they produce black colonies after 3–7 days. This color is related to the bacteria’s ability to assimilate hemoglobin from the medium, which is transformed into protohemin and stored in bacterial cells [7, 9].

*P. gingivalis* rods created many virulence factors to be able to reproduce in the host’s reservoir. Fimbria are the main virulence factor [10]. These are thin, protein structures protruding from the outer membrane of the bacterial cell [7]. According to a study conducted in Japan by Amano et al., *P. gingivalis* produces two types of fimbriae: one consists of a protein encoded by the *fim A* gene, the other of the protein encoded by the *mfa1* gene [11]. Six *FimA* genotypes were found, of which the *FimA II* and *FimA IV* genotype is the most common in patients with periodontitis, while in healthy patients the *FimA I* genotype is most often observed [7, 10]. Despite the difference in the composition of amino acids and in terms of antigens, they perform the same function. They participate in the initial invasion, allowing *P. gingivalis* to adhere to the outer host membrane by adhesion to the cellular integrin α5beta1. As a result, they are more easily absorbed by the host phagocytes and dendritic cells, so they are not subject to immune surveillance by the host [7].

In addition, fimbria has been shown to induce pro-inflammatory cytokines such as IL-1β and IL-6 by CD4 + T helper cells and tumor necrosis factor α (TNF-α) by macrophages [7, 12]. An important factor of virulence after the adhesion of *P. gingivalis* is the production of biofilm in the form of plaque [9]. The biofilm components provide protection against phagocytosis of the bacterial cell and against the effects of antibiotics [13].

Another factor of virulence of *P. gingivalis* (strain PK1924 Serotype K5) is the capsule composed of glucose, galactosamine, glucosamine and uric acid compounds, and lipopolysaccharide (LPS) present on the outer membrane. It has endotoxin properties. It interferes with the distribution of leukocytes at the site of colonization. The LPS released from disintegrating cells activates macrophages through Toll-like receptors present on their surface [9, 14]. The previously produced biofilm protects *P. gingivalis* against phagocytosis. Macrophages produce cytokines. Neutrophils are activated and inflammation develops at the site of colonization [7]. In addition, LPS causes the inhibition of alkaline phosphatase, α1 collagen and osteocalcin differentiation and mineralization in stem cells of the periodontal ligament, which are involved in the regeneration of periodontal tissues [7, 9]. This mechanism explains the characteristic symptom of chronic periodontitis, i.e. the refraction of the alveolar bone and the surrounding tooth tissues [14].

Another virulence factor are the enzymes produced by *P. gingivalis.* The key enzyme enabling the growth of this bacteria, in the oral cavity, is proteases. This enzyme produces two types of proteases: serine proteases and cysteine proteases, so-called gingipain that cleaves the C-terminus polypeptide within arginine (gingipain R) or lysine (gingipain K). These enzymes degrade extracellular matrix proteins, such as fibronectin and collagen [7]. They impair the host defense mechanism by destroying immunoglobulins, complement components C3 and C5, and a-defensin derived from neutrophils [9, 12]. Increased extracellular production of the enzyme exceeds the activity of serine protease inhibitors, which leads to the continuous destruction of infected tissue. The free amino acids released from the disintegrating host proteins are used by *P. gingivalis* for the synthesis of its own proteins and growth [15]. In addition, gingipain decomposes fibrinogen and heme proteins, which allows bacterial growth through availability of hemin [7]. It has been shown that gingipain K has an additive effect on *Tanerella forsythia* growth in the biofilm of *P. gingivalis* [16].

## Characteristics of *Tannerella forsythia*

*Tannerella Forsythia* is a Gram-negative, anaerobic bacterium. Described by Tanner and co-workers, it was referred to as Bacteroides forsythus. Currently, it belongs to the genus *Tanerella* [17]. The bacteria’s breeding is not easy due to its demanding growth conditions.

Microbial complexes were identified in the subgingival plaque by Sigmund Socransky. *T. forsythia* along with *P. gingivalis and T. denticola* belong to the so-called “Red complex”. Development of *T. forsythia* along with other periodontal pathogens in the oral cavity may cause gingivitis and lead to periodontal disease [18]. The presence of *T. forsythia* is associated with an increased risk of periodontitis. The self-mutilating host’s immune response to bacteria and a number of virulence factors of *T. forsythia* [19] contribute to the development of periodontal diseases. In addition, recent studies show that obese and overweight people are more likely to experience periodontal disease, because these people have greater colonization of *T. forsythia* compared to people with normal body weight [17].

Bacteria from the genus *T. forsythia* have many virulence factors. Studies in animal models have shown that it was found to cause cutaneous abscesses in rabbits and mice, as well as alveolar bone defects in mice and rats. In addition, these studies showed a synergistic effect of *T. forsythia* along with other pathogens, e.g. *Fusobacterium nucleatum* or *P. gingivalis*, enhanced the formation of abscesses in rabbits and mice [20]. A synergistic effect occurred in relation to bone loss of the alveolar ridge after oral infection with bacteria forming together with *T. forsythia* “red complex” [17].

*T. forsythia* does not have the ability to break down sugars. To grow and get energy, it needs peptides that are broken down by trypsin-like proteases and cysteine-like proteases PrtH. Trypsin-like protease is involved in the degradation of smaller peptides and in itself does not play a central role in bacterial virulence. It is a serine protease, described for the first time by Grenier. In contrast, the PrtH protease has the ability to cleave larger protein substrates. Initially, it was identified as a detachment factor, called the forsythium detachment factor, due to the participation in cell separation and in the disintegration of the subgingival tissue of the host. The PrtH protein has cytopathic activity that arrests cells in the G2 phase [17]. It affects the reduced adherence of periodontal cells, and also affects the immune system and stimulation of inflammation by inducing the production of interleukin 8 [21].

The next known *T. Forsythia* protease is carilysin. This enzyme may contribute to the dissemination of soluble, active tumor necrosis factor - TNFα from macrophages. It inhibits the activation of complement pathways and causes the degradation of the LL-37 antimicrobial peptide [21]. The calcium-dependent serine protease - mirolase has a similar effect. In addition to the degradation of the LL-37 antimicrobial peptide, it also degrades fibrinogen and hemoglobin [22]. It also has a high ability to modulate the activity of the complement system. It can inhibit the classic and lectin pathway of complement activation. Also, it releases the C5a peptide, which results in the migration of neutrophils [23]. Due to the irreversibility of proteolysis and the mechanism of action of proteases on host cells, their role in pathogenesis of periodontal diseases is indicated. They can cause degradation of protein components of infected tissues, protect bacteria against immune response, and facilitate colonization of pathogens [17, 22].

Another factor of *T. forsythia* virulence is serpin protein, otherwise known as miropin. This protein inhibits serine proteases from neutrophils, thus protecting the bacteria against proteolytic effects of neutrophils [18]. S-glycoproteins associated with the surface layer of bacteria that enable it to adhere to the epithelium also contribute to the pathogenesis. The bacterial surface is also covered by BspA proteins rich in leucine repeat (LRR domain). BspA binds to extracellular fibronectin and fibrinogen, which allows *T. forsythia* adhesion and proliferation. Bacterial surface-exposed lipoproteins are crucial for the growth of bacteria in the host organism. These fractions lead to apoptosis of human gingival fibroblast cells by activating caspase-8 and inducing cytokine formation, e.g. IL-6. *T. forsythia* glycosidase activity allows it to grow and survive. They produce exo-α-sialidase, α-D-glucosidase and N-acetyl-β-D glucosaminidase. These glycosidases hydrolyze the host’s oligosaccharides and proteoglycans to provide the nutrients for *T. forsythia*. In addition, in the presence of glucose, the bacteria accumulates large amounts of toxic methyl glyoxalic product, which contributes to the damage of the hosts tissues. *T. forsythia* has also been shown to increase number of *P. gingivalis* by its ability to reduce fumarate to succinate (precursor of lipid and phospholipid synthesis). *P. gingivalis*, due to its high proteolytic activity, supplies *T. forsythia* peptides and amino acids that are released from damaged host tissues [17]. These pathogens are of key importance in the development of periodontal disease, and there are reports that they may contribute to the development of esophageal cancer.

## Carcinogenesis and types of esophageal tumors

Carcinogenesis is a multi-stage, long-lasting process that causes the development of cancer. Mutations occurring in this process contribute to the destabilization of the cytoskeleton and loss of adhesiveness to neighboring cells. These changes occur under the influence of carcinogenic factors, which may be determined by bacterial species [24]. Carcinogenesis consists of three stages: initiation, promotion and progression. In the initiation phase, a mutation occurs that is irreversible and is transmitted to daughter cells. Morphologically, manifested by hyperplasia or dysplasia. In the next stage, promotion, accumulation of genetic and epigenetic changes takes place. They lead to the conversion of a mutated cell into a cancer cell. Such a cell undergoes uncontrolled divisions and is characterized by a low ability to differentiate. At the promotion stage, the cells are characterized by increased mobility, invasiveness and the loss the integrity with neighboring cells. The last stage is progression, in which cancer cells acquire the ability to infiltrate tissues and to metastasize. They are insensitive to regulatory factors. Their growth is independent of growth factors and hormones. Angiogenesis begins. Cancer cells secrete autocrine growth factors and peptides that stimulate other healthy cells to produce agents that increase the invasiveness of cancer cells [25].

Two histological subtypes differentiate within the esophageal cancer: esophageal squamous cell carcinoma (ESCC) and esophageal adenocarcinoma (EAC). In 50% of cases, ESCC develops in the middle part of the esophagus, while EAC is most often located in the lower part of the organ [26].

There are several stages to the development of esophageal squamous cell carcinoma. The accumulation of genetic changes initiates the process of carcinogenesis, which causes histological changes in epithelial cells [26, 27]. In addition, the presence of chronic inflammation of the mucosa of the esophagus leads to the formation of intraepithelial neoplasia, which is considered a pre-cancerous lesion [28]. In endoscopic examination, this is hardly visible. These changes can be clearly seen by using Lugol’s liquid, which will only stain normal multilamellar flat epithelium [27]. In the microscopic examination, intraepithelial neoplasia is characterized by a disorder of the multilamellar flat epithelial cell system and cytological changes, such as enlargement and hyperchromicity of the nucleus and increased mitotic activity of the cells. The next stage of development is early squamous cell carcinoma. At this stage, cancerous lamina of the esophageal mucosa and submucosa of the esophagus invade through the clone of tumor cells. In advanced esophageal squamous cell carcinoma, metastases to lymph nodes are characteristic and tumor cells may infiltrate the muscle membrane of the esophagus [10].

The development of adenocarcinoma is associated with gastroesophageal reflux disease. As a result of the exposure of the distal epithelium of the esophagus to refluxed gastric and bile acids, intestinal metaplasia occurs, and consequently Barrett’s esophagus. This is the precursor change of EAC [29, 30]. Furthermore, gastro-oesophageal reflux, in addition to direct damage to the esophagus, is responsible for the production of reactive oxygen species. Free radicals cause DNA damage, and the resulting mutations initiate the cancer process [30].

Another factor leading to esophageal adenocarcinoma is obesity. This pathological mechanism is associated with long-lasting inflammation caused by adipocyte hypertrophy. The result may be cell hypoxia, which leads to infiltration of adipose tissue by activated macrophages M2. These cells induce a proinflammatory state releasing cytokines, such as interleukin 6 (IL-6) and tumor necrosis factor α (TNFα) [30].

One of the common factors predisposing to the development of esophageal cancer is the disruption of the gastrointestinal tract microbiome. The presence of the bacteria *Tannerella forsythia* and *Porphyromonas gingivalis* is of key importance. These pathogens are mainly responsible for periodontal disease, however, they are also associated with the development of esophageal cancer [30]. From the research conducted in New York, with 122,000 participants lasting 10 years, Peters et al. confirmed the influence of the oral microflora on the development of esophageal cancer. The results of the study indicate the relationship between *T. forsythia* and adenocarcinoma, while the presence of more *P. gingivalis* is associated with a higher risk of esophageal squamous cell carcinoma [31].

These microorganisms cause interference with the epidermal barrier of the host and induce inflammation, including activation of NF-κB (nuclear factor kappa-light-chain-enhancer of activated B cells) [30]. The pathological mechanism of esophageal cancer with the involvement of *T. forsythia* and *P. gingivalis* will be discussed in detail in the next chapter.

## *Tannerella forsythia* and *Porphyromonas gingivalis* in the ethiopathogenesis of esophageal cancer

Currently, there are a number of documented relationships between bacteria and the development of cancer of a given organ. It is worth noting, however, that this is not a common phenomenon. The most well-known example of this relationship is the role of *Helicobacter pylori* and its contribution to the development of stomach cancer. Other notable examples may be: linking *Salmonella typhi* with gallbladder cancer, *Streptococcus bovis* with developing colorectal cancer, *Chlamydophila penumoniae* with lung cancer, *Propionibacterium acnes* with prostate cancer or *Escherichia coli* with colorectal cancer [32] Recent studies have more and more often pointed to the link between inflammation periodontal disease, and the general state of health and the potential contribution of microorganisms to the development of esophageal cancer. Various forms of periodontal disease caused by *P. gingivalis* and *T. forsythia* indicate the systemic inflammatory response in the body [32, 33]. The long-term presence of *P. gingivalis* in the mouth can infect epithelial cells in the mouth, disrupt the cell cycle and immune responses of the host, as well as cell apoptosis. These are pro-tumor mechanisms in relation to oral squamous cell carcinoma. Due to the high probability of infection of the esophagus from the oral niche, it is possible that infection with *P. gingivalis* may be associated with the development of esophageal squamous cell carcinoma [32].

After exposure to the pathological agent, tissue hormones are released that can destroy the cell membrane of the lysosomes. The lytic enzymes released from lysosomes destroy cellular organelles. Hydrolysis of the ester membrane phospholipids by hydrolases results in the formation of free fatty acids, including the release of arachidonic acid. It is a precursor to numerous eicosanoids, regulating the function of most organs in the human body. Synthase of cyclic prostaglandin peroxide catalyzes the transformation of arachidonic acid into prostanoids, including prostaglandins. Among them, PGD2, PGE2 and PGF2 have the highest activity. The emerging prostaglandins induce inflammation [34].

In the emergence and maintenance of inflammation, cyclooxy genase 2 (COX-2) plays an important role. Literature reports indicate a positive correlation between cyclooxygenase 2 products and the development of tumor and metastatic sites. Activation of COX-2 is influenced by pro-inflammatory cytokines and stress factors. The COX-2 protein is encoded by the COX-2 gene located on chromosome 1. Expression of the COX-2 gene is stimulated by factors involved in the inflammatory reaction, such as: interleukin 1 (IL-1), alpha tumor necrosis factor alpha (TNF-α), the presence of which can be generated by *T. forsythia*, liposaccharides, transcription factors and oncogenes. Over-expression of the COX-2 gene occurs in many cancers, including head and neck cancer. It is also worth noting that overexpression of COX-2 inhibits apoptosis. By inhibiting programmed death, cancer-transformed cells are not degraded and may continue to divide. Over-expression of COX-2 also contributes to tumor neo-angiogenesis. The formation and growth of new blood vessels is directly stimulated by various products, amongst them prostaglandins E2. PGE2 affects the production of vascular endothelial growth factor (VEGF), which is also expressed in many cancers. In addition, PGE2 stimulates tumor cell divisions, angiogenesis and modulates the immune system in such a way that cancer development remains “latent”. In addition, PGE2 receptors are mediators of the metastasis process, and their high expression in tumor cells is indicative of aggressiveness and severity of the disease [35]. The pathway of the E2 prostaglandin also increases the formation of CSCs (cancer stem cells) which are pluripotent. They have the ability to self-renew, differentiate and develop neoplasia. The Notch signaling pathway, in which the signal transduction takes place, is also a significant contributor to the CSC population. It plays a role in differentiating and conditioning the fate of cells. The Notch receptors on cells interact with transmembrane signals on subsequent cells. Certain groups of genes are activated by the signaling cascade [36]. Inflammatory cytokines produced by the inflammation induced by *P. gingivalis* and *T. forsythia* may affect the regulation of the Notch pathway [37].

Another potential mechanism leading to the development of esophageal cancer is matrix metalloproteinases (MMPs). They appear as a result of the action of *Porphyromonas gingivalis* cilia on host cells. These are zinc-dependent proteolytic enzymes. Produced and secreted in the form of an inactive proenzyme. Activation occurs in the extracellular environment. It consists in the proteolytic cleavage of the N-terminal region of the propeptide resulting in the disruption of the bond between the thiol group of the cysteine ​​residue and the zinc ion in the catalytic domain [38, 39]. The main activators of MMP’s are: plasmin, serine proteases and free radicals [40]. Optimal conditions of enzymes activity are a neutral or slightly acidic environment [25]. The enzyme activity is controlled by their endogenous inhibitors, i.e. α-2-macroglobulin, and tissue inhibitors of matrix-derived metalloproteinases (TIMP). α-2-macroglobulin binds MMP irreversibly, and the resulting complex is recognized by scavenger receptors and removed by endocytosis. TIMP has the ability to bind the active form and proenzyme in a reversible manner [41]. Under physiological conditions they are engaged in angiogenesis, embryogenesis and platelet aggregation [40]. Metalloproteinases also play an important role in the development of esophageal squamous cell carcinoma.

**Fig. 1:**
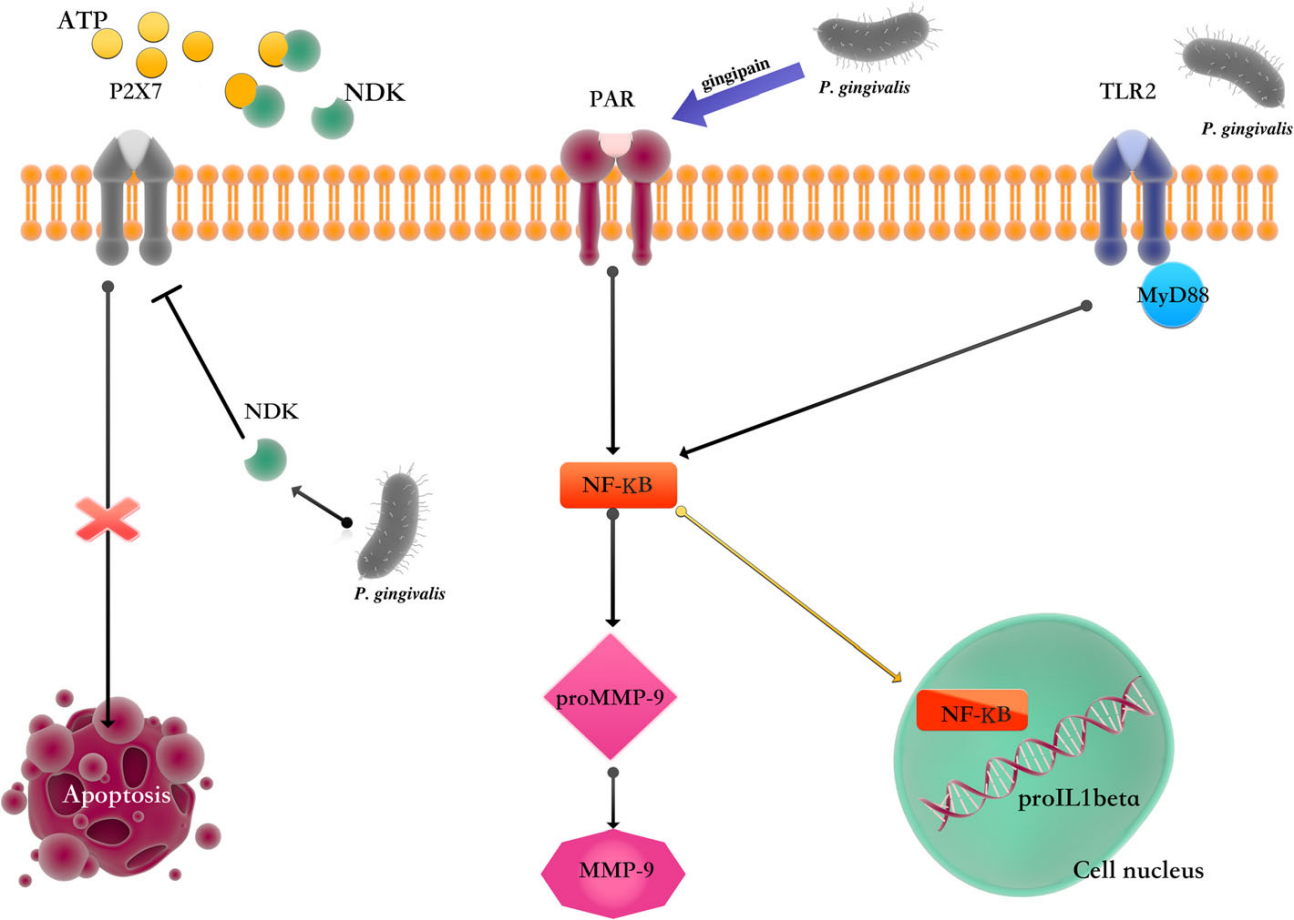
The influence of *P. gingivalis* on the processes of esophageal cancer carcinogenesis

MMP2 and MMP9 are the most active. They belong to the gelatinase group. They are produced by cancer cells, but MMP9 are also produced by neutrophils. The substrate for both enzymes is type IV collagen, and for MMP9, additionally, laminin and fibrillar proteins. They lose their physiological functions when the balance between the activity of MMP-2, MMP-9 and the expression of their TIMP-2 and TIMP-1 inhibitors is disturbed. Then their action is associated with the destruction of the basement membrane and components of the extracellular matrix, such as fibronectin, laminin, collagen and proteoglycans. As a result of damage to the barrier, tumor cells leave the lumen of the vessel. Due to the migration of tumor cells, metastases develop [25, 40]. In addition, MMP9 significantly affects the activity of cytokines and chemokines. The ability to chemokine degradation plays a special role in the process of cancer. MMP9 inactivating chemokines produced by cancer cells inhibits the inflow of neutrophils to the place of inflammation and, consequently, the development of esophageal cancer [25, 41]. In addition, *P. gingivalis* has the ability to secrete nucleoside kinases (NDK). They catalyze the transfer of orthophosphates from nucleoside triphosphates, such as ATP, to nucleoside diphosphates. Consequently, the pathogen inhibits the ATP-dependent activation of caspase-1 and maturation of IL-1β, as well as the reduction of P2X7 receptor activity responsible for the induction of apoptosis [42]. The discussed mechanisms are presented in Fig. 1.

*P. gingivalis* produces gingipain, which activates the Protease Activated Receptor (PAR), leading to the production of MMP-9 metalloproteinases, and the conversion of proMMP-9 to MMP-9. The nucleoside diphosphate kinases (NDK) capture ATP by decreasing the activation of the pro-apoptotic P2X7 receptor.

Bacteria also have an indirect effect on the nourishment of tumor cells involving GLUT-1 and GLUT-4 transporters. A tumor formed as a result of uncontrolled proliferation of cancer cells has an increased nutritional demand for oxygen and glucose. Insufficient amount of nutrients leads to transformation of aerobic metabolism into anaerobic. Cancer cells extract energy from the breakdown of ATP bonds that comes from the glycolysis process and the Krebs cycle. Under anaerobic conditions, the resulting pyruvate is then converted to lactic acid in the fermentation process. The energy gain of anaerobic glycolysis is only 2 ATP molecules, and in aerobic conditions we get 30 ATP molecules from one cycle. However, for rapidly dividing cancer cells, this is not very important because their main goal is to acquire building components for the development of daughter cells [43, 44]. From research carried out by Warburg in the 1920’s, it was proved that tumors have the ability to conduct anaerobic glycolysis even under aerobic conditions [45]. This process is more beneficial for them. Due to this process, proliferating cells take up larger amounts of glucose, which is the source of building components. *T. forsythia* and *P. gingivalis* are responsible for the overexpression of GLUT 1 and GLUT4 through the induction of TNF-alpha signaling pathways and reactive oxygen species (ROS). ROS are generated as a result of the inhibition of electrons transported to oxygen and suppression of oxidative phosphorylation [44].

Glucose uptake by tumor cells is carried out using glucose transporters defined as GLUT. The over-expression of GLUT-1 and GLUT-4 is of particular importance. The GLUT-1 transporter is the most common isoform among the family of these proteins. In addition to cancer cells, it is present in erythrocytes, where it accounts for 3–5% of their membrane proteins, as well as in the placenta, eye and endothelium of the blood-brain barrier. It consists of 492 amino acids and is encoded by the SLC2A1 gene [43]. The GLUT-4 transporter is encoded by the SLC2A4 gene. It is found in skeletal muscle, cardiomyocytes and adipose tissue cells. In addition to glucose, it also transports dehydroascorbic acid [46].

Overexpression of GLUT-1 and GLUT-4 transporters has been observed in many types of tumors and correlates with the increase in tumor invasiveness [43, 46]. It has been suggested that inhibition of these proteins reduces the rate of glucose uptake, induces cell cycle arrest, and consequently, the arrest of tumor cell proliferation in vitro and in vivo [45]. Thus, the inhibition of GLUT transporters could be applied in the treatment of cancer by inhibiting its further development. However, it would be difficult, because the proteins in question are not specific only to cancer cells. Until now, such measures have not been taken in the treatment of cancer.

## Prevention

The major etiological factors such as alcohol and smoking are well studied. Eradication of these factors and early diagnosis are the most desirable prevention. However, as we discussed above, bacteria play a crucial role in etiopathogenesis of esophageal cancer. Professor Ahn and co-workers from NYU Langone Health’s Perlmutter Cancer Center in New York investigated during the decade 122, 000 patients and their oral microbiota. They found that the presence of *T. forsythia* was linked with higher risk of EAC, and the presence of *P. gingivalis* was connected to higher risk of ESCC. Moreover, professor Ahn pointed that Streptococcus and Neisseria were linked to a lower risk of esophageal cancer [31].

Study provided by Abnet in China (2001) on 28,868 participants, also reported that poor oral hygiene was associated with significantly elevated risk of developing esophageal cancer, gastric cardia cancer and gastric non-cardia cancer [47].

Sepehr et al. observed higher risk of esophageal squamous dysplasia in group of 124 patients in northeastern Iran. He noticed that risk increased gradually with poorer degrees of oral health [48].

## Summary

Both *Tannerella forsythia* and *Porphyromonas gingivalis* play an important role in establishing and developing esophageal cancer. An interesting question seems to be why, in spite of the common occurrence of these species of bacteria, only some people have cancerous changes in the esophagus. Partial response is the effect of other bacteria that constitute the individual flora of the oral cavity. One should also take into account the multifactorial aetiology of cancer development and, in this respect, consider the share of bacteria as a significant, but not the most important factor.

Detection of *P. gingivalis* and *T. forsythia* in pre-cancer lesions may become an important element of cancer diagnostics and become a prognostic indicator of esophageal cancer.

Improving oral hygiene and treatment of periodontitis can significantly reduce the occurrence of esophageal, head and neck cancer. In addition, virulence factors such as adhesive *FimA.* play an important role in the transformation of normal cells into cancer cells and can become a potential target for new anti-cancer therapies.

## Abbreviations

CSCs: Cancer stem cells
EAC: Esophageal adenocarcinoma
ESCC: Esophageal squamous cell carcinoma
GCO: Global Cancer Observatory
GERD: Gastroesophageal reflux disease
GLUT: Glucose transporter
IL-1β: Interleukin 1 β
IL-6: Interleukin 6
LPS: Lipopolysaccharide
MMP: Matrix metalloproteinase
NDK: Nucleoside diphosphate kinases
PAR: Protease activator receptor
PGE2: Prostaglandin E2
ROS: Reactive oxygen species
TNF-α: Tumor necrosis factor
